# The Clinical and Pathological Features of Children With Microscopic Polyangiitis

**DOI:** 10.3389/fped.2021.645785

**Published:** 2021-04-15

**Authors:** Qian Li, Li-Chun Yu, Feng-Xia Li, Jing Wang, Yuan Chen, Shu-Zhen Sun

**Affiliations:** ^1^Department of Pediatric Nephrology and Rheumatism and Immunology, Shandong Provincial Hospital Affiliated to Shandong First Medical University, Jinan, China; ^2^Department of Pediatric Nephrology and Rheumatism and Immunology, Shandong Provincial Hospital Affiliated to Shandong University, Jinan, China

**Keywords:** children, microscopic polyangiitis, clinical pathology, female, prognosis

## Abstract

**Objective:** The aim of this study was to explore the clinical features, pathological characteristics, and the prognosis of children with microscopic polyangiitis (MPA).

**Methods:** Ten children with MPA that were hospitalized in our hospital were included in this study. The children's pre-diagnosis status, clinical manifestations, renal pathology, treatment, and prognosis data were analyzed retrospectively.

**Results:** All 10 cases included female patients with a median age of 8.9 years old at the time of diagnosis. MPO-ANCA antibody was positive in all cases, combined with a positive anti-GBM antibody in two cases. Nine cases had primary AAV and one had antithyroid drug (ATD)-associated MPA (secondary to methimazole). Renal involvement was found in all 10 patients, lung impairment was present in eight cases, and anemia was present in nine patients. Renal biopsies were performed in all 10 patients. Segmental focal or global glomerular necrosis was observed in 70% of the patients (7/10). The treatment mainly included steroid use combined with Cyclophosphamide and Mycophenolate. The follow-up s of the patients revealed normal renal function in eight patients and progression to end-stage renal disease (ESRD) in two patients.

**Conclusions:** Female predisposition and positive MPO-ANCA antibody were prominent in children with MPA. The patients' kidneys and lungs were the most frequently involved organs. Corticosteroid combined with immunosuppressive therapy was recommended for the treatment of MPA. Early diagnosis, prompt aggressive treatment, and regular follow-ups are also very important factors associated with a good prognosis.

## Introduction

Microscopic polyangiitis (MPA) belongs to the antineutrophil cytoplasmic antibody (ANCA) associated vasculitis (AAV). AAV is the necrotizing inflammation of the small and medium vessels and is characterized by anti-neutrophil cytoplasm autoantibody related to a group of diseases. It is also a kind of systemic vasculitis involved with the small vein, mall artery, and blood capillary. MPA typically exhibits multiple organ involvement including the patients' kidneys and lungs, which are the most commonly affected organs. MPA is also considered to occur mostly in adults and rarely in children. Previous reports have shown that AAV disease types are significantly different in different regions and races; GPA was mainly seen in Nordic and British Caucasian AAV patients. However, in southern Europe, Japan, and China, MPA is dominant. This is especially found in China where MPA patients account for the majority (80%) (1). Contrary to adults, female children with AAV have a higher incidence rate than males and their clinical manifestations and treatment programs are similar to adults, but the long-term prognosis is better than in adults (2). Children with MPA are also more likely to be females and the prevalence reaches a peak in early puberty (3, 4). In 2016, it was described in the EMA (the European Medicines Agency) standard of the AR Chi Ve investigation network that among the largest pediatric MPA cohort to date (48 patients), females accounted for 73% of the patients and had an average age of 10.8 years old (5). From 2003 to 2013, the first affiliated hospital of Sun Yat-Sen University diagnosed 20 children with MPA without GPA or EGPA, with a male to female ratio of 4:12 and an average age of 8.9 years old (6).

In this study, the clinical characteristics and pathological features of pediatric patients with MPA were retrospectively analyzed in order to explore the clinical features, pathological characteristics, and the prognosis of children with MPA.

## Materials and Methods

### The Selection of Patients

Ten female pediatric patients with MPA, aged ≤ 14 years and diagnosed in Shandong Provincial Hospital Affiliated to Shandong First Medical University between January 2000 and December 2018 were enrolled in this study. Their clinical and pathology data were retrospectively analyzed. Patients with MPA who had received the Antithyroid Drug (ATD) were defined as ATD-associated MPA and the other patients were defined as primary MPA (7). The diagnosis of primary MPA was based on the 2012 revised Chapel Hill Conference Nomenclature of Vasculitides (8). All patients were treated according to the same protocol.

The current study was approved by the ethical committee of our hospital and all patients' legal guardians signed written informed consent.

### The General Data of the Patients

Renal pathology was performed in all 10 cases and repeated renal biopsy was performed in one case after 2-year treatment.

Disease activity was scored according to the Birmingham Vasculitis Activity Score (BVAS).

### The ANCA Analysis

Serum samples of the patients were screened for ANCA by antigen-specific enzyme-linked immunosorbent assay (ELISA) for PR3 or MPO. Serum samples of the patients were screened for PR3-ANCA, MPO-ANCA, anti-GBM and anti-dsDNA by antigen-specific enzyme-linked immunosorbent assay (ELISA). Serum ANA levels were tested by Indirect Immunofluorescence Assay. Serum levels of CRP, C3 and C4 were measured by immune turbidimetry. The changes of erythrocyte sedimentation rate (ESR) were determined by Westergren method.

### Renal Pathology

A renal biopsy was performed on all 10 patients. The renal biopsy specimens were examined by both immunofluorescence and light microscopy and electron microscopy. The immune complex deposition was defined as a score of 0 to 4+ in staining for any kind of immunoglobulins (Igs) observed by immunofluorescence microscopy and/or electron-dense deposits observed by electron microscopy. All patients biopsied after the same amount of disease duration.

### Treatment Protocols

The induction therapy consisted of Prednisone (1–2 mg/kg per day) plus Cyclophosphamide (CTX, 8–12 mg/kg per day, 2 days, once every 2 weeks, the cumulative dosage <150mg/kg). Patients with severe necrotizing crescentic glomerular nephritis and pulmonary hemorrhage were also treated with Methylprednisone (MP, 7.5–15 mg/kg per day, 3 days) pulse therapy. For maintenance therapy, low doses of Prednisone combined with immunosuppressive drugs such as CTX and Mycophenolate Mofetil (MMF) were administered.

### Treatment Response

Complete remission (CR) is defined as (9): (1) The normalization of renal function if renal insufficiency is present; and (2) The disappearance of hematuria, proteinuria, and extra renal manifestations of systemic vasculitis.

Partial remission (PR) is defined as (9): (1) The stabilization or improvement of renal function if renal insufficiency is present; (2) Dialysis independent if renal failure is present; and (3) The resolution of hematuria and proteinuria and/or the resolution of extra renal manifestations of systemic vasculitis.

Treatment failure is defined as (9): (1) The progressive decline in renal function with persistent active urine sediments or; (2) Persistence or the new appearance of any extra-renal manifestations of vasculitis or; (3) Death.

## Results

### The Clinical and Renal Pathological Features

All 10 patients were female and were diagnosed at the age of 5–13 years old, with a median age of 8.9 years old. As shown in Table 1, all 10 patients had extra renal involvement manifestations: 90% of the patients (9/10) had moderate to severe anemia. Eighty percentage of the patients (8/10) had respiratory tract symptoms, such as cough or hemoptysis, abnormal pulmonary X-ray or CT examination, presenting a patchy, nodular grainy image, localized emphysema or increased and blurred lung texture. A rash that was presented as purpura was found in 20% of the patients (2/10). Renal involvement with hematuria and proteinuria was observed in all 10 children. Gross hematuria was seen in 30% of the patients (3/10). Seventy percentage of the patients (7/10) showed mild to moderate proteinuria and 30% of the patients (3/10) showed nephrotic proteinuria. Impaired renal function was seen in 60% of the patients (6/10). Only one patient had Hashimoto's thyroiditis. Other patients have no other diseases. None of them have a family history of autoimmune diseases. Nine of the 10 cases were diagnosed as primary AAV. The other 1 case included a history of hyperthyroidism and received a Methiomidazole administration and was diagnosed as ATD-associated MPA (case 9). The time from onset (time provided by the chief complaint) to diagnosis ranged from 1 week to 3 years, with an average of 4.5 months (Table 1).

**Table 1:** Summary of clinical features of 10 children with MPA.

**Patient**	**Gender**	**Age, yrs**	**Disease course, mos**	**Organ involvement**	**Extrarenal involvement**	**Pulmonary X-ray or CT**	**Renal involvement**	**GFR at Diagnosis**	**BVAS**	**Immunosuppressive Therapy**	**Time of follow-up, yrs**	**Prognosis**
1	F	5	36	Lung, Kidney	Fever, weakness, weight loss, lung damage, anemia	Multiple patchy glass grinding	Gross hematuria, massive proteinuria	9.66	19	MPP	5.4	ESRD
2	F	13	1	Gstro-intestinal, Kidney	Fever, weakness, weight loss, abdominalgi, vomiting, anemia	–	Hematuria, mild proteinuria	39	18	MPP+MMF	5.5	Mild proteinuria, PR
3	F	9	3	Skin, Lung, Kidney	Purpura, analgesia, lung damage, anemia	Multiple patchy glass grinding	Hematuria, mild proteinuria	63	19	MPP+CTX+MMF	5.2	Microscopic hematuria, PR
4	F	9	0.25	Lung, Kidney	Abdominalgi, headache, anemia	Double lung texture increased blur	Gross hematuria, massive proteinuria	5.3	16	HD+MPP+CTX+LEF+MMF	9	Mild proteinuria, PR
5	F	10	0.75	Lung, Kidney	Lung damage, anemia	Patches, nodules, ground glass	Hematuria, moderate proteinuria	72.5	23	PE+MPP+CTX+MMF	2.8	Microscopic hematuria, PR
6	F	6	1	Lung, Kidney	Lung damage, anemia	Ground glass, localized emphysema	Gross hematuria, moderate proteinuria	143	16	MPP	2	CR
7	F	6	1	Skin, Lung, Kidney	Rash, oral ulcer, lung damage, anemia	–	Hematuria, mild proteinuria	120	20	MPP+MMF	2	Microscopic hematuria, PR
8	F	12	1	Lung, Kidney	Abdominalgi, lung damage	Patchy density	hematuria	154	21	PRED+MMF	0.5	CR
9	F	13	0.75	Lung, Kidney	headache, oral ulcer, lungdamage, anemia	Patchy high density shadow	Hematuria, mild proteinuria	142	13	MPP+MMF	0.3	Mild proteinuria, Microscopic hematuria, PR
10	F	6	0.75	Lung, Kidney	Abdominalgi, lung damage, anemia	Patchy ground glass	Hematuria, massive proteinuria	5.3	22	MPP+CTX	4.3	ESRD

*BVAS, birmingham vasculitis activity score (at time of diagnosis); HD, hemodialysis; PE, plasma exchange; PRED, prednisone; MPP, methylprednisolone impact; CTX, cyclophosphamide; LEF, leflunomide; MMF, mycophenolate mofetil; ESRD, end stage renal disease; PR, partial remission*.

As shown in Table 2, renal biopsies were obtained in all 10 patients. Case 4 underwent a second renal biopsy after 2 years of the drug administration. The cellular or fibrous crescents were shown in the renal specimens of 80% of the patients (8/10). Segmental focal or global glomerular necrosis was observed in 70% of the patients (7/10). Extensive or focal renal interstitial fibrosis was found in 60% of the patients (6/10). Immunofluorescence staining of Igg, IgA, IGM, C3, C1q, and FRA was performed in all 10 children with renal biopsy; no IC deposits were observed in 30% of the patients (3/10), a small amount of deposition was seen in 40% of the patients (4/10), and granular or linear distribution in the mesangial area or capillary wall was observed in 70% of the patients (7/10).

**Table 2:** Summary of renal pathological features of 10 children with MPA.

**Patient**	**Disease course before renal biopsy, mos**	**No. Glomerular**	**No. Crescents**	**Cellular crescents**	**Fibrocellular crescents**	**Fibrious crescents**	**No. Sclerotic glomeruli**	**Focal/Segmental sclerotic**	**Immune complex**	**Location of Immune complex**	**Interstitial fibrosis**
1	36.25	13	3	3	0	0	9	0	–		80%
2	1.25	15	5	1	2	2	2	2	C3 ++, FRA+	linear distribution in capillary wall	Wide distribution
3	3.25	20	7	4	0	3	10	0	–	–	40%
4	0.5	9	6	6	0	0	0	0	–	–	–
4 (2 years later)	24.5	12	1	0	0	1	1	4	IgA+, IgG+, FRA++, IgM+++, C1q+-++	granular distribution in the mesangial area	Focal distribution
5	1	31	1	0	1	0	2	16	IgM+++	granular distribution in the mesangial area	Focal distribution
6	1.3	12	0	0	0	0	0	3	IgA+, IgM+	granular distribution in capillary wall	Multifocal distribution
7	1.5	20	2	0	2	0	0	0	C3, FRA 3+	granular distribution in capillary wall	–
8	2.5	15	0	0	0	0	0	0	IgA, IgM+	granular distribution in the mesangial area	–
9	0.75	14	3	0	3	0	1	3	IgA, IgM+	granular distribution in capillary wall	–
10	1	38	31	31	0	0	7	0	IgG+++, C1q++	linear distribution in capillary wall	–

*FRA, fibrin related antigen*.

### ANCA and Other Laboratory Results

As shown in Table 3, positive MPO-ANCA and negative PR3-ANCA were observed in 100% of the patients (10/10). Twenty percentage of the patients (2/10) had a positive GBM antibody. ANA was tested positive and DSDNA was negative in 1 child with secondary MPA. Fifty percentage of the patients showed increased CRP and 90% showed elevated ESR levels. Decreased C3 levels were found in 10% of the cases and no abnormal serum C4 levels were seen in all 10 patients. Serum CRP levels were elevated in 5 children with AAV, which were reduced to normal level within 20 days after steroid therapy. ESR values were increased in 9 children with AAV, which recover to normal level within 0.25–24 months after steroid combined with immunosuppressive drugs therapy in seven patients. Decreased C3 was found in patient 4. She was given the combination therapy of hemodialysis, methylprednisone pulse and CTX therapy. Serum C3 level returned to normal 6 months later.

**Table 3:** Summary of laboratory tests of 10 children with MPA.

**Patient**	**MPO (0**~**20RU/ml)**	**PR3 (0**~**20RU/ml)**	**GBM (0**~**20RU/ml)**	**ANA (<1:100)**	**dsDNA (0**~**100RU/ml)**	**ESR (0–20mm/h)**	**Time to recover (months)**	**C3 (0.8–1.8g/l)**	**CRP (0-8mg/l)**	**Time to recover(day)**	**C4 (0.1–0.4g/l)**
1	26.8, After 1 month of treatment, there was a decline, but not to normal, and there was no re-examination	1.52	2.03	<1:100	<10	41	Not examined	1.12	95.7	20d	0.2
2	29.61, After 7 months of treatment, it had decreased to normal, and then increased again, reaching the highest of 70.21	1.96	1.52	<1:100	<10	110	6	1.01	7.5		0.25
3	40.6, Continuously above normal (25.71– 62.38)	2.38	1.23	<1:100	<10	56	24	1.41	15.2	10d	0.31
4	34.75, not examined for change	2.12	3.58	<1:100	<10	34	6	0.434	8.8	5d	0.185
5	25.85, After 10 days of treatment, it returned to normal	0.6	40.35, After 10 days of treatment, the reexamination was normal, and the subsequent repeated reexaminations were normal	<1:100	<10	44	6	1.21	17.8	10d	0.2
6	158.25, After 5 months of treatment, it returned to normal	1.0	6.00	<1:100	<10	27	4	1.03	2.16		0.27
7	177.25, not examined for change	2.12	5.00	<1:100	<10	28	3	1.17	10.4	3d	0.23
8	95.33, After 5 months of treatment, it returned to normal	1.2	4.70	<1:100	28.98	13		1.06	1.38		0.14
9	64.37, Follow-up for 4 months gradually decreased, but still higher than normal	1.5	11.40	1:1000(+)	60.42	57	0.25	1.12	3		0.19
10	37.25, were high after reexaminations. 2 months later, hemodialysis was transferred to peritoneal dialysis, and no changes were detected again	2.06	94.64, were high after reexaminations. 2 months later, hemodialysis was transferred to peritoneal dialysis, and no changes were detected again	<1:100	<10	95	Not examined	0.96	2.4		0.32

### Treatment and Prognosis

All of the 10 children were given immunosuppression and symptomatic supportive treatment. 90% of the patients (9/10) received Methylprednisolone pulse therapy, 40% of the patients (4/10) received Cyclophosphamide pulse treatment, 70% (7/10) received MMF treatment, and 10% (1/10) received leflunomide treatment. Hemodialysis and plasmapheresis were performed on the basis of immunosuppressive therapy (Table 1). The follow-up times of the 10 patients ranged from 4 months to 9 years. Two children presented as treatment failure and progressed to ESRD. Eighty percentage of the patients (8/10) maintained their clinical remission. Among them, two patients had complete remission and 6 had partial remission. At present, 1 of the 8 children with clinical remission had stopped taking corticosteroids and immunosuppressive therapy, three had stopped taking corticosteroids therapy and only take oral mycophenolate ester therapy, and three had oral corticosteroids and mycophenolate ester maintenance therapy. The patients' urine test results were normal in two patients with microscopic hematuria in three patients, mild proteinuria in two patients, and microscopic hematuria and mild proteinuria in two patient (Table 1).

## Discussion

MPA is rare in childhood and its pathogenesis is not fully understood. ANCA can be detected in the serum of most MPA patients, most of which are positive for MPO/PANCA, but a few patients can be positive for PR3/CANCA (10, 11). The pathogenesis of AAVs involves a complex interplay network of helper T (TH; TH-17) cells, antigen-presenting cells (APCs), and various cytokines like IL-23, IL-1β, TNFα, myeloperoxidase (MPO), and proteinase 3 (PR3), as well as the complement system (12, 13). A variety of environmental factors such as infection and drugs are also believed to be related to ANCA production and vasculitis, among which the reported anti-thyroid drugs mainly include Tabazole, Propylthiazine, and Methimazole (14). In this study, 10 cases of children with MPA tested positive for the serum MPO antibody and 2 cases tested positive for the anti-GBM antibody. Nine patients were diagnosed as primary AAV and 1 13-year-old patient was diagnosed with Hashimoto thyroiditis with hyperthyroidism for 4 years. The long-term oral administration of Methimazole was considered as secondary MPA. ANA was negative in all patients with primary MPA but positive in the girl with ATD-associated MPA. One patient had a low C3 serum level,9 patients had an increased ESR, and elevated CRP was found in 5 patients. However, the relationships between disease activity and serum C3, ESR, CRP levels were not observed in this study.

MPA usually affects multiple organs. The lungs are the most frequently involved extra renal organ. MPA is the most common cause of pneumohemorrhagic nephritis syndrome. The common manifestations of lung damage include diffuse alveolar hemorrhage, dyspnea, hemoptysis, cough, chest pain and imaging features are mainly ground glass and patchy shadows (15). In this study, 8 of the 10 MPA patients (80%) had respiratory symptoms, such as cough or hemoptysis and abnormal lung X-ray or CT examination, which manifested as a patchy, nodular grind glass shadow, localized emphysema or increased and blurred lung texture. The kidneys are also most commonly affected in MPA, and may also be the only affected organ. In the AR Chi Ve survey network study, according to the EMA criteria, renal involvement, including proteinuria, microscopic hematuria, and renal insufficiency were present in 3/4 of the patients and serum creatinine levels were moderately to severely elevated in 48% of the children with renal involvement. 1/3 of the children developed hypertension and 1/4 of the children needed renal replacement therapy treatment at the onset (16). In this study, all 10 children had renal impairment, hematuria, and proteinuria, including three cases of gross hematuria, three cases of nephrotic proteinuria, six cases with renal impairment at diagnosis, and two cases of hemodialysis or plasmapheresis.

The pathological features of the kidneys in MPA patients were glomerular capillary segmental fibrinoid necrosis with a crescent formation. Different stages of the glomerular disease were also found in one patient (16). Currently, there are no guidelines for the treatment of MPA or AAV in children and the clinical treatment scheme mainly refers to the recommendations of adult AAV. The 2016 AAV treatment recommendation issued by the European Federation of Rheumatology (EULAR) and the European Dialysis and Transplantation Association (EDTA) stated that glucocorticoid (GC) (including Methylprednisolone pulse) combined with Cyclophosphamide or Rituximab is recommended for AAV-induced remission therapy that threatens organ function and life. For AAV induction and remission therapy without organ function involvement, GC combined with Methotrexate or Mycophenate is recommended (17). In 2017, the expert consensus of the children's blood purification committee of the pediatrician branch of the Chinese Medical Doctor Association stated that plasma exchange can be used in AAV children (18). Early diagnosis and early treatment are key factors for patients with MPA. Although most of the patients responded to induction therapy, some patients progressed to CKD stages.

In our study, the follow-up time of the 10 cases was 4 months to 9 years; 8 cases had a normal renal function and two cases progressed to ESRD.As long as AAV was diagnosed, early and intensive immunosuppressive therapy was used. Our remission rate was 80% and was similar to previous studies (3). At present, one of the 8 children with normal renal function had stopped taking prednisone and immunosuppressive therapy; three patients had stopped taking corticosteroids therapy and received Mycophenolate, and; three patients received oral corticosteroids therapy and Mycophenolate in the maintenance phase. The patients' urine test results were normal in two patients, microscopic hematuria was found in three patients, mild proteinuria in two patients, and microscopic hematuria and mild proteinuria in one patient. Therefore, regular and long-term follow-ups are needed for evaluating the prognosis of children with MPA. However, our limitation is that 4 months is still too short to assess the effect of therapy and disease prognosis. In addition, retrospective nature of the research and the wide range of follow up also limited our study because it is difficult to draw firm conclusions if one patient is followed for 4 months and the other for 9 years.

## Conclusions

Female predisposition and positive MPO-ANCA antibody were prominent in children with MPA. The kidneys and lungs were the most frequently involved organs in MPA. Corticosteroid combined with immunosuppressive therapy was recommended for the treatment of the disease. Take-home messages are early diagnosis, prompt aggressive treatment, and regular follow-ups are very important for improving the patients' long-term outcomes.

## Data Availability Statement

The original contributions presented in the study are included in the article/supplementary material, further inquiries can be directed to the corresponding authors.

## Ethics Statement

The studies involving human participants were reviewed and approved by Shandong Provincial Hospital Affiliated to Shandong First Medical University. Written informed consent to participate in this study was provided by the participants' legal guardian/next of kin.

## Author Contributions

QL and L-CY conceptualized and designed the study, drafted the initial manuscript, and reviewed and revised the manuscript. F-XL, JW, and YC designed the data collection instruments, collected data, carried out the initial analyses, and reviewed and revised the manuscript. S-ZS coordinated and supervised data collection, and critically reviewed the manuscript for important intellectual content. All authors approved the final manuscript as submitted and agree to be accountable for all aspects of the work.

## Conflict of Interest

The authors declare that the research was conducted in the absence of any commercial or financial relationships that could be construed as a potential conflict of interest.

## Abbreviations

MPA: microscopic polyangiitis
ATD: antithyroid drug
ESRD: end-stage renal disease
ANCA: antineutrophil cytoplasmic antibody
AAV: antineutrophil cytoplasmic antibody associated vasculitis
EMA: European Medicines Agency
ELISA: enzyme-linked immunosorbent assay
CTX: Cyclophosphamide
MP: Methyl prednisone
MMF: Mycophenolate Mofetil
CR: Complete remission
PR: Partial remission
MPO: myeloperoxidase.
